# Prostate cancer in male *BRCA1* and *BRCA2* mutation carriers has a more aggressive phenotype

**DOI:** 10.1038/sj.bjc.6604132

**Published:** 2008-01-08

**Authors:** A Mitra, C Fisher, C S Foster, C Jameson, Y Barbachanno, J Bartlett, E Bancroft, R Doherty, Z Kote-Jarai, S Peock, D Easton, The IMPACT and EMBRACE Collaborators, R Eeles

**Affiliations:** 1 Translational Cancer Genetics Team, Institute of Cancer Research, Surrey, UK; 2 Cancer Genetics Unit, The Royal Marsden NHS Foundation Trust, London, UK; 3 Department of Histopathology, The Royal Liverpool University Hospital, Liverpool, UK; 4 CR-UK Genetic Epidemiology Unit, Strangeways Research Laboratories, Cambridge, UK

**Keywords:** prostate cancer, *BRCA1* and *BRCA2*, prostate pathology

## Abstract

There is a high and rising prevalence of prostate cancer (PRCA) within the male population of the United Kingdom. Although the relative risk of PRCA is higher in male *BRCA2* and *BRCA1* mutation carriers, the histological characteristics of this malignancy in these groups have not been clearly defined. We present the histopathological findings in the first UK series of *BRCA1* and *BRCA2* mutation carriers with PRCA. The archived histopathological tissue sections of 20 *BRCA1/2* mutation carriers with PRCA were collected from histopathology laboratories in England, Ireland and Scotland. The cases were matched to a control group by age, stage and serum PSA level of PRCA cases diagnosed in the general population. Following histopathological evaluation and re-grading according to current conventional criteria, Gleason scores of PRCA developed by *BRCA1/2* mutation carriers were identified to be significantly higher (Gleason scores 8, 9 or 10, *P*=0.012) than those in the control group. Since *BRCA1/2* mutation carrier status is associated with more aggressive disease, it is a prognostic factor for PRCA outcome. Targeting screening to this population may detect disease at an earlier clinical stage which may therefore be beneficial.

Prostate cancer (PRCA) is the most commonly diagnosed malignancy in men in the United Kingdom. It is thought to be composed of aggressive and indolent varieties. Indolent PRCA may exist for many years without causing symptoms or shortening life expectancy. Aggressive PRCA may cause symptoms difficult to palliate with conventional treatments and is likely to shorten life expectancy. Distinguishing which men are at risk of which types of disease could have far-reaching consequences not only in the treatment and follow-up of patients but also in the surveillance of groups at risk of aggressive PRCA.

The morphology of breast cancer found in female *BRCA1* and *BRCA2* germline mutation carriers has been studied extensively. The histopathological features of breast tumours from patients with *BRCA1* and *BRCA2* mutations differ from each other and from sporadic breast cancers (Lakhani *et al*, 1998). *BRCA1* and *BRCA2* breast cancers are of higher grade (Lakhani, 2001). *BRCA1*-associated tumours are more likely to be oestrogen, progesterone receptor and Her2 receptor negative, express TP53 protein, and demonstrate medullary/atypical medullary morphology. Lakhani (2001) predicts that a woman diagnosed with high-grade, oestrogen receptor-negative breast cancer before the age of 30 years has a 40–45% chance of harbouring a *BRCA1* mutation, compared with a 4–5% chance if these parameters are not met (Lakhani, 2001). Many of the features identified in breast cancer in women who are *BRCA1* mutation carriers are associated with a poor prognosis. If similar data were available for men with PRCA who are carriers of these mutations, weighing up the options for radical treatment or active surveillance could be simplified with this added information.

Despite studies on the incidence of PRCA in male *BRCA1* and *BRCA2* mutation carriers, there are sparse data recording the histopathology of the disease they develop. The largest and most recent study reports a series of 30 male *BRCA2* (999del5) Icelandic founder mutation carriers. The mutation carriers had PRCA with a significantly higher Gleason score, lower mean age of diagnosis, more advanced stage and shorter median survival than a control group (Tryggvadóttir *et al*, 2007). Before this, the largest series was reported by Giusti *et al* (2003).Twenty-nine carriers of Ashkenazi Jewish (AJ) founder *BRCA1/2* mutations who had developed PRCA were compared with non-carriers with PRCA. No difference was seen in Gleason pattern, incidence of prostatic intraepithelial neoplasia (PIN) or atypical adenomatous hyperplasia (Giusti *et al*, 2003). In another study, Hubert *et al* (1999) compared two groups each of 87 Israeli men: one group diagnosed with PRCA and the other a control group. The number of AJ founder mutations was found to be the same in each group. The three mutation carriers in the PRCA group were found to have an average Gleason score above 8, compared with a Gleason score of 5.9 for non-carriers with PRCA (Hubert *et al*, 1999). The serum PSA level was also higher than that in non-carriers. Grönberg *et al* (2001) reported a family of five male 999del5 *BRCA2* mutation carriers from Sweden, who developed poorly differentiated PRCA at an early age of onset with a shortened life expectancy (Grönberg *et al*, 2001). A recent PRCA screening study enrolled 19 male *BRCA1/2* mutation carriers (12 *BRCA1* and 7 *BRCA2*) and an age-matched control group of men with at least one first-degree relative with PRCA. Annual serum PSA (threshold for prostate biopsy 4 ng ml^−1^) and digital rectal examination were undertaken. The two mutation carriers with PRCA were noted to have Gleason scores of 6 and 9 compared with the single subject from the control group who had a Gleason score of 6 (Horsburgh *et al*, 2005).

When applied correctly, the Gleason scoring system is the most robust and reproducible method currently available for grading the morphological appearances of PRCA (Gleason *et al*, 1966). The criteria and their application are well documented but often require adjustment and standardisation if these criteria have not been applied by an expert urological pathologist (Foster, 1991; Deshmukh and Foster, 1998; Epstein *et al*, 2005; Berney *et al*, 2007a, 2007b). Currently, the Gleason score is used as a prognostic indicator for individual PRCAs in the general population. But the features of PRCA developing in men who harbour a *BRCA1* or *BRCA2* mutation are not well established. This paper reports the histopathological features of the first UK series of male *BRCA1* and *BRCA2* mutation carriers with PRCA.

## MATERIALS AND METHODS

Prostate cancer tissue was collected from cases and controls. A total of 20 cases with *BRCA1* or *BRCA2* mutations was collected from throughout the United Kingdom and Ireland. These were identified from four sources described below: the EMBRACE (Epidemiological Study of Familial Breast Cancer) study, the IMPACT (Identification of Men with a Genetic Predisposition to Prostate Cancer: Targeted Screening in *BRCA1/2* Mutation Carriers and Controls) study, a Cancer Genetics outpatient clinic and a series of young onset PRCAs.

Men with PRCA enrolled in the EMBRACE study had consented to the use of their prostate tissue samples for further research. The hospitals where these men had undergone prostate biopsy, prostatectomy or transurethral resection of the prostate (TURP) sent blocks/slides containing prostate tissue to AM. This material was coded anonymously with a unique study number. Where original haematoxylin and eosin slides were not sent, new ones were cut at The Institute of Cancer Research from the blocks provided. Twelve slides were obtained in this manner from England, Ireland and Scotland.

IMPACT is an international screening study for men unaffected by cancer with a known *BRCA1* or *BRCA2* mutation (and therefore believed to be at increased risk of developing PRCA). One man who was diagnosed with PRCA was recruited from the IMPACT study.

One individual was recruited from the Cancer Genetics outpatient clinic in the Royal Marsden NHS Foundation Trust (RMH).

Six further slides were obtained from The Institute of Cancer Research. A series of 263 men who had PRCA diagnosed under the age of 55 years had previously undergone retrospective *BRCA2* mutation analysis using conformational sensitive capillary electrophoresis, which was then confirmed by sequencing. Prostate tissues from the six men found to have deleterious *BRCA2* mutations were incorporated into the current study (Edwards *et al*, 2003).

Controls were obtained from a data set of 4893 men with a diagnosis of PRCA who had been treated at the RMH between 1985 and 2006. One control was obtained from the Royal Liverpool University Hospital. Men were matched for age (±5 years) wherever possible. The age range was greater than this in four cases (with differences of 7, 9, 9 and 10 years), where it proved difficult to obtain better-matched controls. PSA and disease stage were matched as closely as possible when this information was available from the medical notes. Where PSA or stage was not available, the controls were chosen to have a higher PSA and/or a more advanced stage (Tables 2 and 4). The controls comprised 17 needle biopsies, 2 TURPs and 1 wedge biopsy. Two men from the control group had been tested for and did not harbour any *BRCA2* mutations.

Histopathological slides were reviewed by specialist urological pathologists. Seventeen of the control group PRCA slides and all 20 of the *BRCA1* and *BRCA2* mutation carriers' PRCA slides were reviewed by a single specialist histopathologist from a tertiary referral centre (CSF). Three of the remaining control group slides were reviewed by one other single, specialist histopathologist, CJ from RMH, a specialist tertiary referral centre. The histopathologists each cross-checked the reporting of a subset of the other's tissue sections to ensure consistency.

Throughout this study, we have employed only the original grading system, as described by Gleason (1966) and explained by Professor Deshmukh and Foster (1998) (Gleason, 1966; Deshmukh and Foster, 1998). A poorly differentiated tumour was defined as a Gleason pattern greater than 7, that is, combined Gleason score of 8, 9 or 10. Perineural invasion (PNI) and lymphovascular invasion (LVI) were also recorded in each case. The handling of tertiary grades is of practical importance only with respect to grade 7 cancers. The convention defined in the consensus meeting of the International Society of Urological Pathologists, 2005, in which CSF participated, was followed. In this recommendation, PRCAs with a Gleason score of 3+4 or 4+3 together with a tertiary pattern 5 have their PRCA classified as Gleason score 8 or 9, respectively.

### Statistical methods

*BRCA1/2* mutation carriers were matched with controls for age, PSA and stage of disease to minimise the effect of these factors on the Gleason score comparison. McNemar's test was used to compare the Gleason score (⩽7 *vs* >7) and presence or absence of PNI and LVI between cases and controls (see Tables 6–10 and 11). This test looks for differences between the row and column marginal frequencies. It is based on the idea that if there are really no differences in the Gleason scores of *BRCA1/2* mutation carriers and non-carriers, then any differences in our sample must have arisen by chance, and are equally likely to occur in favour of the cases than of the controls. Therefore, if there were really no differences, we would expect the number of cases with a low Gleason score who are matched to a control with a high Gleason score (*n*=1 in Table 6) to be roughly equal to the number of cases with a high Gleason score who are matched to a control with a low Gleason score (*n*=10 in Table 6).

## RESULTS

Tables 1, 2, 3, 4 and 5 show the characteristics of the patients. The staging of the disease has been corrected to the TNM classification of 2002.

### Patients' characteristics

There was a total of 20 cases and 20 controls (4 *BRCA1* and 16 *BRCA2*) (Tables 1 and 3). For the majority of matched cases and controls, where the cases presented with symptomatic or asymptomatic (screen-detected) disease, the controls were matched in the same way. If it was not possible to match for presentation (screen-detected or symptomatic), the controls were matched to present with symptoms for comparison with cases that presented as a result of screening. This was not possible for three *BRCA2* cases and one *BRCA1* case; however, where a control either had unknown presentation or screen-detected disease, the cases that were matched presented with symptomatic disease (Tables 2 and 4).

Of the *BRCA2* mutation carriers, nine presented with symptoms, which preceded the diagnosis of PRCA, four men had screen-detected disease and this information was missing for three men. Nine of the control group presented with symptoms, four were screened and three had an unknown presentation.

Three *BRCA1* mutation carriers had screen-detected disease.

### Comparison of *BRCA1* and *BRCA2* mutation carriers with PRCA and controls

There was a significant difference in the Gleason score between patients with *BRCA1* and *BRCA2* mutations who had developed PRCA and the controls (Table 6, McNemar's test, *P*=0.012). There was no difference in PNI or LVI between the two groups (Tables 7 and 8, McNemar's test, *P*=1.00 in both cases).

### *BRCA2* patients alone

When the group of *BRCA1*/*2* mutation carriers was divided, the statistically significant difference in Gleason scores between *BRCA2* mutation carriers and controls was maintained (Table 9, *P*=0.016 using McNemar's test). There was again no difference in PNI or LVI in the two groups (Tables 10 and 11, McNemar's test, *P*=1.00 in both cases). The *BRCA1* mutation carrier group was too small to show statistically significant results.

## DISCUSSION

We have reported the histopathology found in the first UK series of male *BRCA2* and *BRCA1* mutation carriers with PRCA. The men who carry the mutations have a diverse heritage (consistent with our population); as such our series is not confined to a few founder mutations. The number of PRCAs with Gleason score 8, 9 or 10 is significantly greater in the *BRCA1/2* mutation carrier group than that in the control group (*P*=0.012). This difference is also seen between the *BRCA2* mutation carriers alone and the matched control group (*P*=0.016). These findings would support studies that suggest that male *BRCA2* mutation carriers who develop PRCA may have a shorter disease-specific life expectancy than men with PRCA in the general population (Sigurdsson *et al*, 1997; Edwards *et al*, 1998, 2005; Tryggvadóttir *et al*, 2007).

The Breast Cancer Linkage Consortium has conducted a large prospective cohort study. It demonstrated that *BRCA2* mutation carriers have a relative risk (RR) of PRCA of 4.65 rising to 7.33 below the age of 65 years and *BRCA1* mutation carriers have an RR of PRCA of 1.82 under the age of 65 years (BCLC, 1999; Thompson *et al*, 2002). Similar data have been recorded in the AJ population (Struewing *et al*, 1997; Warner *et al*, 1999; Giusti *et al*, 2003), although smaller clinical studies have generally not demonstrated an increased frequency of founder *BRCA1/2* mutations among Jewish men with PRCA (Lehrer *et al*, 1998; Hubert *et al*, 1999; Nastiuk *et al*, 1999; Vazina *et al*, 2000).

In the current data set, six men were identified from a previous study conducted at The Institute of Cancer Research, as described in the Materials and Methods section. As these men were selected for mutation analysis following the diagnosis of young onset PRCA, this data set is skewed for young age and so the controls have been matched for age. The median age of PRCA diagnosis in the *BRCA2* mutation carriers is 52.5 years and in the matched control group it is 55.5 years. The difference between these two age groups is not significant, but the age of onset in these groups is considerably less than the average age of onset of PRCA in the UK general population (75 years) (www.statistics.gov.uk). It is possible that PRCA in a young age group may show a different histopathology to that in an older age group. However, the controls were matched for age and were found to have a significantly lower Gleason score. Prostate cancer incidence has been shown to be higher in *BRCA1* and *BRCA2* mutation carriers under the age of 65 years (BCLC, 1999; Thompson *et al*, 2002). If these carriers do develop more aggressive disease at a younger age than the general population, then screening them for PRCA may be a prudent use of resources.

Matching the cases to the control group was not always straightforward. Prostate cancer dedifferentiates over time (Draisma *et al*, 2006). Therefore, it can be difficult to match men who may or may not have had the disease *in situ* for differing lengths of time. To minimise such lead-time bias, the men in the control group were matched for PSA and stage of disease. This produced complications, as adequate data were not always recorded in the patients' medical notes. In cases where relevant data were found to be missing, the controls were chosen with advanced stage and with a serum PSA level higher than 100 ng ml^−1^. This was done in order to bias the control group towards more advanced disease and therefore with potential for dedifferentiation to a more aggressive Gleason pattern. This means that the effect that we have reported in *BRCA1/2* mutation carriers is probably greater in reality.

Similarly, it could be argued that men who present with symptomatic disease could have a more advanced natural history than those men who present with screen-detected disease (Draisma *et al*, 2006). In this series of men, the controls have been carefully chosen so that they have presented with symptoms more often than the *BRCA1/2* mutation carrier group. In the *BRCA2* series, the control group only presented with screen-detected disease in four men, whereas the *BRCA1* mutation carrier cases presented with symptomatic disease once.

A more difficult variable for which to correct is the fact that this series of cases and controls uses data from a variety of sources, including prostate biopsies, TURPs and prostatectomies for comparison. Also, the patients in this series had sextant biopsies (Hodge *et al*, 1989). Reports have suggested that needle biopsy and radical prostatectomy Gleason scores only match in 45% of cases (Bostwick, 1994; Levine *et al*, 1998; King *et al*, 2004). This is likely to result from intraobserver and interobserver variability in Gleason grading and sampling error in taking the biopsy. Ozdamar *et al* (1996) showed that grading error was greatest with well-differentiated (Gleason scores 2–4) tumours. In these cases, the accuracy was only 15% with needle biopsy (Ozdamar *et al*, 1996). Of patients with Gleason scores 5–7 on needle biopsy, 97% were graded correctly. All those with Gleason scores 8–10 on needle biopsy were graded correctly. Deshmukh and Foster (1998) argue that some pathologists do not identify milder forms of grade 3 PRCA and label it instead as grade 2. In this study, no PRCAs were found to have a Gleason score less than 6.

There is also discordance in the reporting of Gleason scores among pathologists (Allsbrook *et al*, 2001a). This is, however, significantly less between specialist urological pathologists (Allsbrook *et al*, 2001b). Both CJ and CSF are specialist urological pathologists. Recently, these criteria have been used to assess a large number of PRCAs in the United Kingdom and to resolve discrepancies (Berney *et al*, 2007a, 2007b). Risk of interobserver variability was further minimised in this study by having CJ review a subset of the cases and controls; there was no discordance between the two histopathologists.

Of the 16 controls for the *BRCA2* series, 14 had not been tested for a *BRCA2* mutation. Edwards *et al* (2003) found that the incidence of *BRCA2* mutation in early-onset PRCA sufferers (under the age of 55 years) could be as high as 3.0% (Edwards *et al*, 2003). The average age of our control group was 55.5 years, so we would expect that at most one of these men could have harboured a *BRCA2* mutation.

In conclusion, this data set is the first UK series of male *BRCA1* and *BRCA2* mutation carriers with PRCA. These mutation carriers have a significantly higher Gleason score than the non-carriers with PRCA. It would follow that the *BRCA1* and *BRCA2* mutations are therefore prognostic markers for aggressive PRCA. If screening for PRCA using serum PSA detects malignant disease at an earlier stage, this may have the potential to reduce mortality from a histologically aggressive disease in this population. These data would support screening male *BRCA1* and *BRCA2* mutation carriers for PRCA. The IMPACT study is currently recruiting nationally within the United Kingdom and internationally in Australia and Norway. IMPACT will investigate whether targeted PSA screening detects PRCA in this subgroup of high-risk men.

## Figures and Tables

**Table 1:** *BRCA2* carrier mutation status

IM01	6819delTG
IM04	6174delT
IM05	5910C>G (Y1894X)
IM06	7771insA
IM07	6503delTT
IM09	3386T>G
IM11	6503delTT
IM12	3386T>G
IM13	7084delAAAAG^*^
IM14	2558insA
IM15	7772insA^*^
IM16	6710delACAA
IM18	Nucleotide variation 2, intron G>C 1 BP BEF splice site
IM19	5531delTT
IM21	8395G>C (D2723H)
IM22	8205-1G>C

These mutations are described as pathogenic in the Breast Cancer Mutation Database (BIC; http://research.nhgri.nih.gov/bic/) with the exception of those marked ^*^ that are described as pathogenic by Edwards *et al* (2003).

**Table 2:** Age (years), PSA (ng ml^−1^), TNM stage, method of detection and year of presentation for *BRCA2* mutation carriers

**Age (years)**		**PSA (ng ml^−1^)**		**TNM stage**		**Detection**		**Year of presentation**	
**Case**	**Control**	**Case**	**Control**	**Case**	**Control**	**Case**	**Control**	**Case**	**Control**
57	59	16.6	7.05	M1	T3aN1M1c	Symptomatic	Symptomatic	1998	1999
58	61	4.7	3.4	M1	T2aNXM1	Symptomatic	Symptomatic	2004	1998
67	67	107	400	Unknown	T3NxM1	Unknown	Symptomatic	2004	2004
54	52	4.6	6.0	Clinical T1c, pT2c	Clinical T1	Screened	Screened, *BRCA2* negative	2000	1996
46	47	151	127	Negative bone scan, clinically localised	Clinical T3N1	Screened	Symptomatic	1998	2001
46	53	4.5	17	M1–ext. iliac LN	Clinical T3NxMx	Screened	Symptomatic, *BRCA2* negative	2004	1995
57	56	4.7	3.5	pT2cN0	Clinical T2a	Symptomatic	Screened	2004	2003
46	55	Predated PSA	203	M1 bone metastases	Clinical T3N0M1	Symptomatic	Symptomatic	1971	2003
47	52	32	49.5	T3N0M1	T3N1M1	Symptomatic	Unknown	1995	Unknown
48	57	<1	4.6	T2N0M0	T1c	Symptomatic	Screened	1992	2003
53	58	227	200	T3aN0M0	T2NxM1	Symptomatic	Symptomatic	1994	1996
52	49	Unknown	141	Unknown	T4NxM1	Symptomatic	Symptomatic	1990	1990
44	39	139	48.5	M1	T4N1M1	Symptomatic	Symptomatic	2000	2005
56	53	Unknown	>100	T3N0M0	T3NxM1	Unknown	Symptomatic	1992	1995
45	50	4.1	5.6	Organ confined	T2, organ confined	Screened	Screened	1997	2002
57	67	685	666	Unstaged	Unstaged	Unknown	Unknown	1999	2006

**Table 3:** *BRCA1* carrier mutation status

IM02	3875delGTCT
IM03	1294del40
IM08	185delAG
IM10	185delAG

These mutations are described as pathogenic in the Breast Cancer Mutation Database (BIC; http://research.nhgri.nih.gov/bic/).

**Table 4:** Age, PSA, TNM stage, method of detection and year of presentation for *BRCA1* mutation carriers

**Age (years)**		**PSA (ng ml^−1^)**		**TNM stage**		**Detection**		**Year of presentation**	
**Case**	**Control**	**Case**	**Control**	**Case**	**Control**	**Case**	**Control**	**Case**	**Control**
62	64	11.6	13	P T3N0M0	T3aN0M0	Screened	Symptomatic	1998	1995
69	68	6.5	6.7	Clinical T3a	T3a	Screened	Symptomatic	2000	1996
70	74	13.4	8.3	Clinical T3N0M0	T3	Symptomatic	Screened	2003	2003
48	50	3.8	0.6	Clinical T1c	T3bN0M0	Screened	Symptomatic	2006	1997

**Table 5:** Age and PSA data available

	**Median age (cases)**	**Median age (controls)**	**Significance**
*BRCA2* series	52.5 (44–67)	55.5 (39–67)	Wilcoxon signed-ranks, *P*=0.056
*BRCA1* series	65.5 (48–70)	66.0 (50–74)	Wilcoxon signed-ranks, *P*=0.141

	**Median PSA (cases)**	**Median PSA (controls)**	**Significance**
*BRCA2* series	24.3 (4.1–685.0)	32.8 (3.4–666.0)	Wilcoxon signed ranks, *P*=0.583
*BRCA1* series	9.1 (3.8–13.4)	7.5 (0.6–13.0)	Wilcoxon signed ranks, *P*=0.465
Combined *BRCA1*+*2*	12.5 (3.8–685.0)	10.7 (0.6–666.0)	Wilcoxon signed ranks, *P*=0.438

**Table 6:** Gleason score of *BRCA1*/2 mutation carriers and their matched controls

	**Controls**		
	**Gleason ⩽7**	**Gleason >7**	**Total**
*BRCA1/2 mutation carriers*			
Gleason ⩽7	2	1	3
Gleason >7	10	7	17
Total	12	8	20

**Table 7:** PNI status of *BRCA1*/2 mutation carriers and their matched controls

	**Control PNI**		
	**No**	**Yes**	**Total**
*BRCA1/2 cases PNI*			
No	5	6	11
Yes	6	2	8
Total	11	8	19^a^

PNI=perineural invasion.

a PNI was reliably commented upon in 19 of the cases.

**Table 8:** LVI status of *BRCA1/2* mutation carriers and their matched controls

	**Control LVI**		
	**No**	**Yes**	**Total**
*BRCA1/2 cases LVI*			
No	5	7	12
Yes	6	1	7
Total	11	8	19^a^

LVI=lymphovascular invasion.

a LVI was reliably commented upon in 19 of the cases.

**Table 9:** Gleason score of *BRCA2* mutation carriers and their matched controls

	**Controls**		
	**Gleason ⩽7**	**Gleason >7**	**Total**
*Cases*			
Gleason ⩽7	2	0	2
Gleason >7	7	7	14
Total	9	7	16

**Table 10:** PNI status of *BRCA2* mutation carriers and their matched controls

	**Control PNI**		
	**No**	**Yes**	**Total**
*Case PNI*			
No	4	5	9
Yes	5	1	6
Total	9	6	15^a^

PNI=perineural invasion.

a PNI was reliably commented upon on 15 of the cases.

**Table 11:** LVI status of *BRCA2* mutation carriers and their matched controls

	**Control LVI**		
	**No**	**Yes**	**Total**
*Case LVI*			
No	4	5	9
Yes	5	1	6
Total	9	6	15^a^

LVI=lymphovascular invasion.

a LVI was reliably commented upon on 15 of the cases.
